# Apparent and standardized ileal digestibility of calcium in wheat and soybean meal for growing quail chicks using direct method

**DOI:** 10.1016/j.psj.2025.104830

**Published:** 2025-01-16

**Authors:** Sima Heydari, Mahmoud Ghazaghi, Mohammad Rokouei, Mehran Mehri

**Affiliations:** Department of Animal Science, University of Zabol, Zabol 98661-5538, Iran

**Keywords:** Calcium, Digestibility, Direct method, Quail, Wheat

## Abstract

Calcium (Ca) is necessary for bone health and metabolic functions in poultry, however, the extent to which it can be utilized varies among feed ingredients. The goal of this study was to determine the apparent ileal digestibility (AID) and standardized ileal digestibility (SID) of calcium in wheat and soybean meal (SBM) in young quail chicks using a direct method. Three dietary treatments were used in the experiment: a calcium-free basal diet to determine endogenous calcium losses, and two diets with either wheat or SBM as the primary calcium sources. Titanium dioxide was incorporated as an indigestible marker for precise measurement of ileal digestibility. On day 30, a total of 300 male quail chicks were weighed and then randomly assigned to one of the three treatments. Each treatment comprised five replicate pens, with 20 birds per pen. On day 34, the birds were euthanized using carbon dioxide, and ileal digesta samples were collected from the distal two-thirds of the ileum to analyze Ca content. The endogenous ileal Ca loss was determined to be 179 ± 68.6 mg/kg of dry matter intake (DMI), providing a baseline for calculating SID values. The AID of calcium for wheat grain was determined to be 24.3 %, with the SID measuring 31.2 %. However, SBM had higher Ca digestibility, with AID and SID values at 34.6 % and 39.9 %, respectively. Understanding digestibility metrics is crucial for optimizing dietary formulations to ensure adequate Ca intake, particularly when plant-derived sources of Ca are used.

## Introduction

Calcium (Ca) plays a crucial role in quail nutrition, particularly for skeletal development, egg production, and various physiological functions. In growing quail, Ca is essential for proper bone formation and growth, with studies suggesting that quail require approximately 0.65-0.90 % Ca in their diet during the early growth stages (Perine et al., 2016, 2022). For laying quail, Ca is vital for eggshell formation and quality, with requirements increasing significantly during the egg-laying phase up to 2.68-3.16 % for optimal egg production and shell quality (Amoah et al., 2012; Stanquevis et al., 2021). Calcium also contributes to muscle contraction, nerve impulses, enzyme activation, and hormone secretion in the body. Adequate Ca intake is necessary to prevent skeletal weakness and alterations in egg production. However, it's important to note that both Ca deficiency and oversupply can negatively impact quail performance, highlighting the need for precise Ca management in quail nutrition (Perine et al., 2016, 2022).

The measurement and application of Ca digestibility in dietary sources for poultry has gained significant importance in recent years, as it provides a more accurate assessment of the Ca available to the bird compared with total Ca content. The traditional use of total Ca in feed formulation can lead to oversupply, which has been shown to adversely affect fat utilization, metabolizable energy, and the availability of other minerals (David et al., 2023). Moreover, excess Ca can interfere with phosphorus (P) absorption and utilization, a critical concern given the environmental and economic implications of P in poultry diets (David et al., 2023). By shifting towards a digestible Ca system, similar to the digestible P system already in use, nutritionists can more accurately meet the bird's requirements while minimizing potential negative impacts on performance and nutrient excretion (David et al., 2023; Pelicia et al., 2009).

This study aimed to determine the apparent ileal digestibility (AID) and standardized ileal digestibility (SID) of calcium (Ca) in wheat and soybean meal (SBM) in growing Japanese quail from 30 to 34 days posthatch using the direct method.

## Materials and methods

### Ethics statement

The protocol for this study was approved by the Research Animal Ethics Committee at the University of Zabol and adhered to the standards set by the Iranian Council of Animal Care. All experimental procedures were conducted following the ARRIVE guidelines, and the National Institutes of Health (NIH) standards for animal research (Du Sert et al., 2020).

### Chemical analyses

Feed ingredients including corn, wheat, and SBM, were analyzed for dry matter using a forced-air oven (Model UF110, Memmert) at 105°C for 24 h (DM; method 930.15, AOAC, 2006), ash content was measured in a muffle furnace (Model F46130, Thermo Scientific) at 550°C for 4 h (method 942.05, AOAC, 2006), crude fiber was analyzed using an Ankom Fiber Analyzer (Model A200, Ankom Technology) (method 978.10, AOAC, 2006), ether extract was determined using a Soxhlet extractor (Model SER 148, Velp Scientifica) with petroleum ether as the solvent (method 2003.05, AOAC, 2006), Ca content was quantified using an atomic absorption spectrophotometer (Model AA-7000, Shimadzu) (method 934.01,AOAC (2006), and crude protein content was determined using a Kjeldahl apparatus (CP; method 990.03, AOAC, 2006). Titanium dioxide (TiO₂) content was determined using the spectrophotometric method described by (Short et al., 1996).

### Birds management and experimental diets

A total of 300 Japanese quail chicks (mixed sex) were used in a digestibility assay from 30 to 34 d posthatch to measure the AID and SID of Ca in wheat and SBM. From hatching until 29 d of age, quail chicks were fed a standard diet formulated to meet the nutrient requirements of growing Japanese quails, based on NRC (1994) recommendations. The temperature and humidity of the experimental room were maintained at 22 ± 1.50 °C and 60 ± 2.55 %, respectively. The lighting program was 18L:6D throughout the experiment. Three dietary treatments composed of a Ca-free diet (Ca content of corn was less than 0.012 % Ca), wheat, and SBM diets were developed and each treatment had 5 replicates and 20 birds in each replicate pen. A corn-based Ca-free diet (mash form) was formulated to measure the ileal endogenous Ca losses (IECaL), and two experimental diets were formulated, each containing either wheat or SBM as the sole source of Ca (Table 1). Titanium dioxide as the indigestible marker was added to the diet (5 g/kg). At the end of the experiment, all the birds were euthanized by CO_2_ asphyxiation to collect ileal digesta from the distal two-thirds of the ileum by gently flushing with distilled water into plastic containers. Digesta from birds in each pen were pooled and then stored at −20°C until they were analyzed further. Performance (feed intake, body weight, and gain: feed) of the birds was measured during the experiment.

**Table 1:** Composition and nutrient characteristics of experimental diets.

Ingredient	Amount (%)		
	Ca-free diet	Wheat	Soybean meal
Test ingredient	-	97.12	45.00
Corn, grain	97.00	-	51.08
Soybean oil	1.00	1.00	2.55
NaH_2_PO_4_	-	-	0.05
KHCO_3_	0.62	0.57	-
TiO_2_	0.50	0.50	0.50
NaCl	0.32	0.30	0.31
Mineral Premix^1^	0.25	0.25	0.25
Vitamin Premix^2^	0.25	0.25	0.25
NaHCO_3_	0.06	0.01	-
Nutrient composition			
AME (kcal/kg)	3337	2807	3000
CP (%)	7.68	13.6	25.9
Lysine (%)	0.23	0.39	1.43
Methionine (%)	0.16	0.18	0.37
Methionine + Cysteine (%)	0.33	0.44	0.77
Threonine (%)	0.28	0.36	0.99
Tryptophan (%)	0.06	0.17	0.31
Ca (%)	0.02	0.05	0.14
Available P (%)	0.10	0.11	0.20
Na (%)	0.16	0.16	0.16
K (%)	0.91	1.00	1.05
Cl (%)	0.23	0.23	0.23
DEB (mEq/kg)^3^	79.2	269	270

1 Mineral premix provided per kilogram of diet: Mn (from MnSO4·H2O), 65 mg; Zn (from ZnO), 55 mg; Fe (from FeSO4·7H2O), 50 mg; Cu (from CuSO4·5H2O), 8 mg; I [from Ca (IO3)2·H2O], 1.8 mg; Se, 0.30 mg; Co (from Co2O3), 0.20 mg; Mo, 0.16 mg.

2 Vitamin premix provided per kilogram of diet: vitamin A (from vitamin A acetate), 11,500 IU; cholecalciferol, 2,100 IU; vitamin E (from dl-α-tocopheryl acetate), 22 IU; vitamin B12, 0.60 mg; riboflavin, 4.4 mg; nicotinamide, 40 mg; calcium pantothenate, 35 mg; menadione (from menadione dimethylpyrimidinol), 1.50 mg; folic acid, 0.80 mg; thiamine, 3 mg; pyridoxine, 10 mg; biotin, 1 mg; choline chloride, 560 mg; ethoxyquin, 125 mg.

3 Dietary Electrolyte Balance: represents dietary Na +*K* − Cl in mEq/kg of diet. AME and amino acid contents were determined in our laboratory (not published).

### Calculations

Following Anwar et al. (2016), the AID coefficient of Ca (AIDC) was calculated using the formula below, which uses the indigestible marker ratio between diets and digesta:

$$\text{AIDC}=1-[(Ti_{D}/Ti_{I})\times (Ca_{I}/Ca_{D})]$$

where AIDC represents the apparent ileal digestibility coefficient of Ca, Ti_D_ is the titanium concentration in the diet, Ti_I_ is the titanium concentration in the ileal digesta, Ca_I_ is the Ca concentration in the ileal digesta, and Ca_D_ is the Ca concentration in the diet. All analyzed concentrations were expressed in grams per kilogram of DM.

Ileal endogenous Ca losses (IECaL; g/kg DM intake) were calculated by the following formula:

$$\text{IECaL}=Ca_{O}\times \left(Ti_{I}/Ti_{D}\right)$$

where Ti_D_ is the titanium dioxide concentration in the diet, Ti_I_ is the titanium concentration in the ileal digesta, Ca_O_ is the Ca concentration in the ileal digesta.

Standardized ileal digestibility coefficients of Ca (SIDC) of the test diets were then calculated as follows:

$$\text{SIDC}=\text{AIDC}+\left[\text{IECaL}\left(g/\text{kg}\text{of}\text{DMI}\right)/Ca_{D}\left(g/\text{kg}\text{of}\text{DM}\right)\right]$$

where AIDC and SIDC represent the apparent ileal digestibility and standardized ileal digestibility coefficients of Ca, respectively, while IECaL represents the ileal endogenous Ca losses (g/kg of DMI) and Ca_D_ represents the Ca concentration in diet (g/kg of DM).

### Statistical analysis

The data were analyzed using a one-way ANOVA in the General Linear Model (GLM) procedure of SAS (2002), with pen means as the experimental unit. Statistical significance was set at *P* < 0.05, and significant differences between means were determined using the Least Significant Difference (LSD) test.

## Result and discussion

**Fig. 1:**
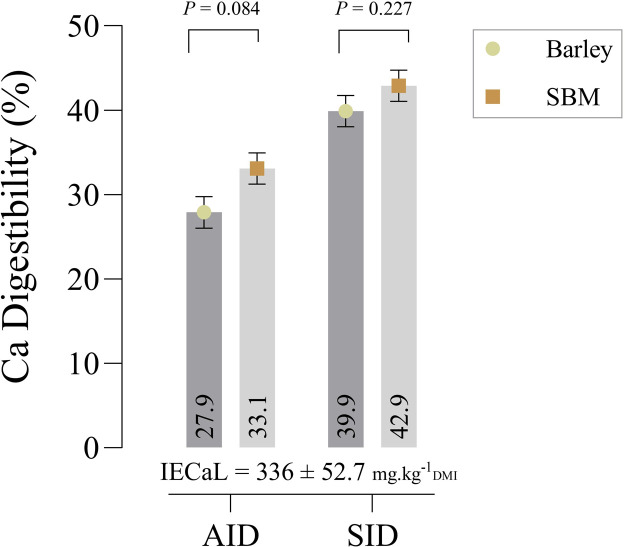
Apparent ileal digestibility (AID), standardized ileal digestibility (SID), ileal endogenous losses (IECaL) of calcium in growing quail chicks.

The AIDC in wheat was 0.243, which was lower than that in SBM (0.346; *P* = 0.044; Fig. 1). In contrast, the SIDC in wheat (31.2 %) and SBM (39.9 %) did not show significant difference (*P* = 0.081), and IECaL was estimated at 179 ± 68.6 mg/kg of DMI.

The present study assessed the AIDC and SIDC in wheat and SBM for growing quails using a direct method. These results are discussed in the context of previous studies on Ca digestibility in poultry, particularly broiler chickens, due to absence of such data on growing quail chicks. The comparison of IECaL between quail and broiler chickens reveals notable distinctions, which could be attributed to species-specific physiological and dietary differences. In our research, IECaL in growing quail was measured at 179 mg/kg of DMI. This value falls within the range observed in broilers, albeit at a slightly lower level than some values reported in recent studies. For broilers, IECaL varies significantly depending on age, diet composition, and experimental methodologies. David et al. (2021) reported IECaL values of 236 mg/kg DMI in broiler growers, which is higher than the value observed in quail in the current study. Similarly, Anwar et al. (2016) found IECaL values in broilers ranging from 88 to 292 mg/kg DMI, highlighting variability due to feed types and measurement methods. Anwar and Ravindran (2020) also noted that IECaL values in broilers can vary widely, from 125 to as low as 46 mg/kg DMI, depending on diet composition and assay methodology. Such data imply that dietary components influence endogenous calcium losses, making direct species comparisons complex. The lower IECaL values seen in quail compared to broiler growers might reflect differences in growth rate and calcium metabolism, as well as the unique digestive physiology of quail, which are smaller and have different nutrient requirements (Cullere et al., 2018; Emadinia et al., 2020; Rodehutscord and Dieckmann, 2005). This comparison underscores the need for species-specific calcium requirement assessments in poultry nutrition to optimize feed formulations for growth and bone health.

The AIDC for wheat was significantly lower than that for SBM, indicating that calcium in wheat is less digestible than in SBM. However, when corrected for endogenous losses (i.e., by estimating SIDC), the differences between wheat and SBM became non-significant. This suggests that the actual bioavailability of calcium from both ingredients is similar when endogenous losses are accounted for, aligning with previous studies that report variability in calcium digestibility across different feed ingredients. For example, Lee and Kong (2024) noted that plant-based ingredients like SBM tend to have higher SID values for Ca than cereal grains like wheat in broiler chickens. The lower AIDC observed for wheat may be attributed to the presence of anti-nutritional factors such as fiber and phytates, which can interfere with Ca absorption (Sultan et al., 2024). It is important to note that while wheat contains anti-nutritional factors, SBM is also likely higher in phytate content compared to wheat. This difference in phytate content could have implications for the observed results, as phytates are known to bind Ca and reduce its bioavailability.

This outcome underscores the significance of correcting for endogenous losses in nutrient digestibility research, as it can change the interpretation of results. Endogenous losses of Ca, which are the Ca lost from the gastrointestinal tract that is not derived from the dietary source, are an essential factor to consider when evaluating the standardized availability of Ca. Without accounting for these losses, the apparent digestibility values may overestimate or underestimate the actual Ca that is available for absorption by the bird (David et al., 2021). The disappearance of the differences in Ca digestibility between wheat and SBM after correcting for endogenous losses suggests that quails may absorb Ca from both ingredients with similar efficiency when the confounding effects of basal endogenous losses are removed. This finding is consistent with studies of broilers, such as Zhang and Adeola (2018), who observed that once endogenous losses are considered, the digestibility of Ca from various sources becomes more comparable across feed ingredients. Moreover, Lee and Kong (2024) highlighted that SID coefficients provide a more accurate measure of Ca bioavailability, as they adjust for variations in endogenous losses that can obscure true nutrient absorption efficiency. In line with these findings, it is clear that correcting for endogenous Ca losses provides a more accurate reflection of the true digestibility of Ca from different feed ingredients. This emphasizes the importance of considering endogenous losses in future studies of Ca digestibility in quail, as they can significantly alter the interpretation of AIDC data and offer a clearer picture of the nutritional value of feed ingredients. Therefore, while AIDC values may differ between ingredients, such differences may not be as pronounced when the standardization method is applied, particularly in species like quail, where endogenous losses might be more significant in some scenarios. In our recent study, the AID and SID of Ca in SBM were 33.1 % and 42.9 %, respectively (Khajali et al., 2025), while in the present study, those values were 34.6 % and 39.9 %. These results highlight the consistent higher calcium digestibility of SBM in young quail chicks, aligning with its known nutritional profile. Compared to SBM, the AID and SID values for barley and wheat were lower, but differences in endogenous calcium losses and experimental design may explain the variation in the standardized values across these feed ingredients.

In conclusion, the correction for IECaL through the estimation of SIDC eliminated the differences observed in AIDC between wheat and SBM. While AIDC values indicated a lower Ca digestibility in wheat (24.3 %) compared to SBM (34.6 %), the SIDC values (31.2 % for wheat and 39.9 % for SBM) were not significantly different, highlighting the importance of adjusting for endogenous losses. These findings demonstrate that accounting for endogenous losses provides a more accurate measure of Ca bioavailability and suggests that the Ca digestibility of wheat and SBM is comparable when standardized methods are used, offering valuable insights for more precise dietary formulations in growing quails. However, it is important to note that other factors, such as ingredient processing, variability in anti-nutritional factors, and interactions with other dietary components, might also influence the observed results, as the numeric differences between wheat and SBM remain noteworthy.

## Funding statement

This research was supported by the University of Zabol. The funding body had no role in the design of the study, data collection and analysis, interpretation of results, or preparation of the manuscript.

## Declaration of competing interest

The authors declare that they have no conflicts of interest regarding the publication of this article.

The authors whose names are listed immediately below certify that they have NO affiliations with or involvement in any organization or entity with any financial interest (such as honoraria; educational grants; participation in speakers’ bureaus; membership, employment, consultancies, stock ownership, or other equity interest; and expert testimony or patent-licensing arrangements), or non-financial interest (such as personal or professional relationships, affiliations, knowledge or beliefs) in the subject matter or materials discussed in this manuscript.
